# Patients with immune-mediated inflammatory diseases receiving cytokine inhibitors have low prevalence of SARS-CoV-2 seroconversion

**DOI:** 10.1038/s41467-020-17703-6

**Published:** 2020-07-24

**Authors:** David Simon, Koray Tascilar, Gerhard Krönke, Arnd Kleyer, Mario M. Zaiss, Franz Heppt, Christine Meder, Raja Atreya, Entcho Klenske, Peter Dietrich, Abdullah Abdullah, Thorsten Kliem, Giulia Corte, Harriet Morf, Moritz Leppkes, Andreas E. Kremer, Andreas Ramming, Milena Pachowsky, Florian Schuch, Monika Ronneberger, Stefan Kleinert, Clara Maier, Axel J. Hueber, Karin Manger, Bernhard Manger, Carola Berking, Matthias Tenbusch, Klaus Überla, Michael Sticherling, Markus F. Neurath, Georg Schett

**Affiliations:** 1 Department of Internal Medicine 3, Friedrich-Alexander University (FAU) Erlangen-Nuremberg and Universitätsklinikum Erlangen, Ulmenweg 18, 91054 Erlangen, Germany; 2 Deutsches Zentrum fuer Immuntherapie (DZI), FAU Erlangen-Nuremberg and Universitätsklinikum Erlangen, Ulmenweg 18, 91054 Erlangen, Germany; 3 Department of Dermatology, FAU Erlangen-Nuremberg and Universitätsklinikum Erlangen, Ulmenweg 18, 91054 Erlangen, Germany; 4 Department of Internal Medicine 1, FAU Erlangen-Nuremberg and Universitätsklinikum Erlangen, Ulmenweg 18, 91054 Erlangen, Germany; 5 Institute of Biochemistry, Emil-Fischer-Zentrum, FAU Erlangen-Nuremberg, Fahrstraße 17, 91054 Erlangen, Germany; 6 Department of Orthopedic and Trauma Surgery, FAU Erlangen-Nuremberg and Universitätsklinikum Erlangen, Krankenhausstraße 12, 91054 Erlangen, Germany; 7 Rheumatology Clinical Practice Erlangen, Möhrendorferstraße 1c, 91056 Erlangen, Germany; 8 Institute of Clinical and Molecular Virology, FAU Erlangen-Nuremberg and Universitätsklinikum Erlangen, Schlossgarten 4, 91054 Erlangen, Germany; 9 Rheumatology Section, Sozialstiftung Bamberg, Buger Straße 80-82, 96049 Bamberg, Germany; 10 Rheumatology Practice Bamberg, Hainstraße 6, 96047 Bamberg, Germany

**Keywords:** Crohn's disease, Viral infection, Epidemiology, Rheumatoid arthritis

## Abstract

Immune-mediated inflammatory diseases (IMIDs) of the joints, gut and skin are treated with inhibitors of inflammatory cytokines. These cytokines are involved in the pathogenesis of coronavirus disease 2019 (COVID-19). Investigating anti-SARS-CoV-2 antibody responses in IMIDs we observe a reduced incidence of SARS-CoV-2 seroconversion in IMID patients treated with cytokine inhibitors compared to patients receiving no such inhibitors and two healthy control populations, despite similar social exposure. Hence, cytokine inhibitors seem to at least partially protect from SARS-CoV-2 infection.

## Abstract (web summary)

Cytokine storm seems to be a common feature of severe COVID-19 pathology. Here, the authors show a reduced rate of SARS-CoV2 positivity in a large population of patients with immune-mediated inflammatory diseases if they are already being treated with cytokine or JAK inhibitors, indicating these treatments are safe to continue and are possibly protective against COVID19.

## Introduction

In December 2019, first cases of human infection by a new coronavirus, named Severe Acute Respiratory Syndrome Corona Virus 2 (SARS-CoV-2), were reported in Wuhan leading to a cluster of respiratory infections^1^. This coronavirus disease 2019 (COVID-19) rapidly spreads worldwide and the virus increasingly challenges patient groups with enhanced infection risk. The severity of COVID-19 is highly heterogeneous and is modulated by yet to be identified host factors. While most individuals experience no or rather mild respiratory symptoms, COVID-19 can also lead to severe pneumonia with acute respiratory distress syndrome and death^2^. Age, smoking (which increases expression of ACE2, the epithelial receptor for SARS-CoV-2) and certain comorbidities, such as diabetes mellitus, arterial hypertension, and obstructive lung diseases, are associated with severe courses of COVID-19^2^.

In the context of COVID-19, patients with IMIDs are of particular interest, as these patients are characterized by intrinsic immune dysfunction as well as immune modulatory treatments that may enhance infection risk. IMIDs often affect the inner and outer barriers of the body such as the joints (rheumatoid arthritis, spondyloarthritis), gut (inflammatory bowel disease), and skin (psoriasis)^3^. Therapeutic interventions for IMIDs target inflammatory cytokines, such as TNF- α, IL-6, and IL-17 that are both involved in the physiological and pathological host response elicited by SARS-CoV-2^4^. We therefore questioned whether IMID patients receiving cytokine inhibitors might be at higher risk for SARS-CoV-2 infection.

In this study, we analyze the prevalence of anti-SARS-CoV-2 immunoglobulin G (IgG) antibodies in patients with IMIDs receiving cytokine-blocking treatments and show that these patients have a lower seroprevalence of SARS-CoV-2 than healthy individuals.

## Results

### Cohorts and anti-SARS-CoV-2 IgG testing

We documented respiratory and other infectious symptoms as well as social behavior during the outbreak of COVID-19 in Europe in February up to April 2020 for a period of 12 weeks. This analysis was performed in patients with IMIDs receiving continuous cytokine blockade for their underlying disease (*N* = 534), in patients with IMIDs receiving no cytokine inhibition (*N* = 259), in health care professionals involved in the treatment of these patients (health-care [HC] control, *N* = 285) and in a cohort of healthy participants unrelated to health care^5^; (non-health care [NHC] control, *N* = 971) from the same region. Details of IMID patients and the control cohorts are depicted in Table 1. To test for exposure to SARS-CoV-2 we made use of recent insights into the anti- SARS-CoV-2 immune response: Spike proteins and the nucleocapsid are known as the main antigens of Corona viruses^6,7^. The S1 domain of the SARS-CoV-2 spike protein has been shown to provide good specificity with limited cross-reactivity to antibodies raised during infections with the four endemic coronaviruses^8^. We therefore used an S1 domain-based antibody ELISA for the initial testing and confirmed all positive samples by determining the antibody response to the SARS-CoV-2 nucleocapsid (N) protein.

**Table 1:** Demographic and clinical characteristics.

	Non-healthcare control	Healthcare control	IMIDs cytokine INH	IMIDs non-cytokine INH
	*N* = 971	*N* = 285	*N* = 534	*N* = 259
**Demographic characteristics**				
Age, mean ± SD, years	43.2 ± 14.3	40.3 ± 12.7	48.9 ± 15.7	55.3 ± 16.1
Females, *N* (%)	274 (28.2)	189 (66.3)	285 (53.4)	152 (58.7)
BMI, mean ± SD	26.5 ± 6.0	23.6 ± 4.4	26.4 ± 5.8	26.4 ± 4.5
Smoking, *N* (%)	181 (18.6)	35 (12.3)	94 (17.6)	40 (15.4)
Diabetes, *N* (%)	59 (6.1)	12 (4.2)	42 (7.9)	14 (5.4)
Hypertension, *N* (%)	117 (12.0)	8 (2.8)	145 (27.2)	75 (29.0)
Chronic lung diseases, *N* (%)	67 (6.9)	7 (2.5)	46 (8.6)	16 (6.2)
**Type of IMID**				
SpA, *N*	0	0	117 (21.9)	34 (13.1)
RA, *N*	0	0	130 (24.3)	106 (40.9)
IBD, *N*	0	0	176 (33.0)	14 (5.4)
Psoriasis, *N*	0	0	63 (11.8)	28 (10.8)
Other^a^, *N*	0	0	48 (9.0)	77 (29.7)
**Treatment**				
TNF Inhibitors, *N* (%)	0	0	227 (42.5)	0
IL-6 Inhibitors, *N* (%)	0	0	44 (8.2)	0
IL-23 Inhibitors, *N* (%)	0	0	85 (15.9)	0
IL-17 Inhibitors, *N* (%)	0	0	51 (9.6)	0
JAK Inhibitors, *N* (%)	0	0	39 (7.3)	0
Others^b^, *N* (%)	0	0	88 (16.5)	0

*BMI* body mass index, *IBD* inflammatory bowel disease, *IL* interleukin, *IMID* immune-mediated inflammatory diseases, *INH* inhibitor, *JAK* Janus kinase, *RA* rheumatoid arthritis, *SpA* spondyloarthritis, *TNF* tumor necrosis factor

^a^Systemic lupus erythematosus, primary Sjogren’s syndrome, systemic sclerosis, polymyositis, IgG4-related disease, sarcoidosis, juvenile idiopathic arthritis, adult onset Still’s disease, periodic fever syndromes, Behcet’s disease, autoimmune hepatitis, giant cell arteritis, takayasu arteritis, granulomatosis with polyangiitis, polymyalgia rheumatica.

^b^Abataceptra, anakinra, apremilast, belimumab, canakinumab, etrolizumab, mepolizumab, rituximab, vedolizumab.

### Prevalence of anti-SARS-CoV-2 IgG in IMID patients

**Fig. 1:**
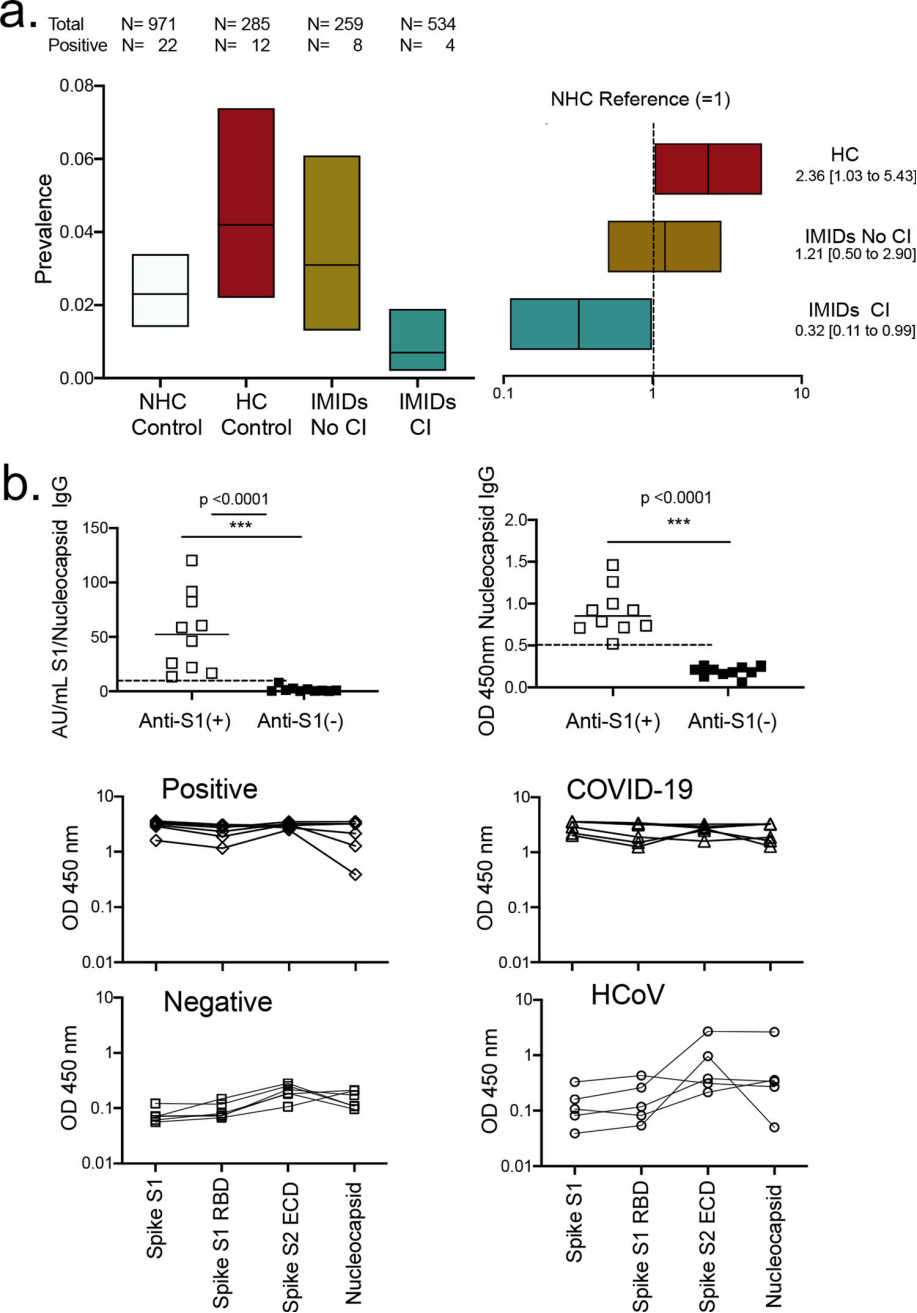
Prevalence of anti-SARS-CoV-2 IgG antibodies across study groups and validation of the results. **a** Left: Prevalence and 95% confidence intervals of a positive anti-SARS-CoV-2 IgG antibody test recognizing the S1 domain of the spike protein in the non-health care (NHC) control cohort, health care (HC) control cohort and immune-mediated inflammatory diseases (IMIDs) with and without cytokine inhibitors (CI); right: risk ratios and 95% confidence intervals of anti-SARS-CoV-2 IgG antibody positivity in the HC control cohort and IMIDs with and without cytokine inhibitors (CI) with the NHC control cohort as reference; **b** Comparison of anti- SARS-CoV-2 IgG positive (anti-S1+; *N* = 10) and negative (anti-S1; *N* = 10) samples (in Euroimmune ELISA) for reactivity in the chemi-luminescent anti- SARS-CoV-2 Spike S1/nucleocapsid IgG test (Yhlo Biotech) and anti-nucleocapsid IgG antibody ELISA (Immundiagnostik Inc). **c** Validation with in-house ELISA testing reactivities against^1^ the S1 domain of the spike protein^2^, the receptor binding domain (RBD) of the S1 domain of the spike protein^3^, extracellular domain (ECD) of the S2 domain of the spike protein and^4^ the nucleocapsid in anti- SARS-CoV-2 IgG negative (*N* = 6) and positive (*N* = 6) samples (in Euroimmune ELISA), COVID-19 patients with positive viral RNA test (*N* = 6) and patients with endemic human coronavirus (HCoV) infection (*N* = 5) in the pre- SARS-CoV-2 time.

Anti-SARS-CoV-2 IgG defined as an OD 450 nm of ≥0.8 in the IgG antibody test against the spike protein domain S1 was found in 2.27% (95%CI 1.42–3.43%) of the NHC control cohort (Fig. 1a). Age-, sex- and, sampling date- adjusted prevalence of anti-SARS-CoV-2 IgG was significantly higher (Poisson model RR 2.36, 95%CI 1.03–5.43; *p* = 0.043 by Poisson regression model) in the HC control cohort (prevalence 4.21%, 95%CI 2.18–7.35%), representing participants from the clinics and practices treating IMID patients. Unexpectedly, only 4 out 534 IMID patients (prevalence 0.75% 95%CI 0.20–1.92%) developed anti-SARS-CoV-2 IgG responses. In the age-, sex- and, sampling-date- adjusted Poisson model prevalence of anti-SARS-CoV-2 IgG responses was significantly lower (RR 0.32, 95%CI 0.11–0.99; *p* = 0.048 by Poisson regression model) compared with reference NHC control group, despite that all of these patients received continuous treatment with cytokine blocking agents. In contrast, IMID patients receiving no cytokine blockade had similar (RR 1.21, 95%CI 0.50–2.90; *p* = 0.676 by Poisson regression model) prevalence of anti-SARS-CoV-2 IgG responses compared with the HC control cohort (prevalence 3.09%, 95%CI 1.33–6.09%). Details on the breakdown of seropositivity stratified by group, age, sex, and other covariates are shown in Table 2.

**Table 2:** Seropositivity stratified by group, age, sex, and comorbidity.

Chararcteristic, *N* (%)	SARS-CoV2 IgG	Non-healthcare control	Healthcare control	IMIDs cytokine INH	IMIDs non-cytokine INH
Age					
≤19	Negative	47 (97.9)	1 (100.0)	5 (100.0)	2 (100.0)
	Positive	1 (2.1)	0 (0.0)	0 (0.0)	0 (0.0)
20–39	Negative	334 (97.9)	141 (97.9)	152 (98.7)	44 (97.8)
	Positive	7 (2.1)	3 (2.1)	2 (1.3)	1 (2.2)
40–59	Negative	461 (97.9)	109 (94.0)	228 (100.0)	93 (96.9)
	Positive	10 (2.1)	7 (6.0)	0 (0.0)	3 (3.1)
60–79	Negative	101 (97.1)	22 (91.7)	136 (98.6)	99 (98.0)
	Positive	3 (2.9)	2 (8.3)	2 (1.4)	2 (2.0)
≥80	Negative	6 (85.7)	–	9 (100.0)	13 (86.7)
	Positive	1 (14.3)	–	0 (0.0)	2 (13.3)
Sex					
Male	Negative	681 (97.7)	87 (92.6)	245 (98.8)	102 (97.1)
	Positive	16 (2.3)	7 (7.4)	3 (1.2)	3 (2.9)
Female	Negative	268 (97.8)	184 (97.4)	284 (99.6)	147 (96.7)
	Positive	6 (2.2)	5 (2.6)	1 (0.4)	5 (3.3)
Diabetes					
No	Negative	837 (98.0)	191 (95.5)	447 (99.3)	204 (96.7)
	Positive	17 (2.0)	9 (4.5)	3 (0.7)	7 (3.3)
Yes	Negative	56 (94.9)	11 (91.7)	41 (97.6)	14 (100.0)
	Positive	3 (5.1)	1 (8.3)	1 (2.4)	0 (0.0)
Hypertension					
No	Negative	780 (98.0)	194 (95.1)	347 (99.4)	144 (96.0)
	Positive	16 (2.0)	10 (4.9)	2 (0.6)	6 (4.0)
Yes	Negative	113 (96.6)	8 (100.0)	143 (98.6)	74 (98.7)
	Positive	4 (3.4)	0 (0.0)	2 (1.4)	1 (1.3)
Chronic lung disease					
No	Negative	829 (98.0)	195 (95.6)	440 (99.1)	203 (97.1)
	Positive	17 (2.0)	9 (4.4)	4 (0.9)	6 (2.9)
Yes	Negative	64 (95.5)	6 (85.7)	46 (100.0)	15 (93.8)
	Positive	3 (4.5)	1 (14.3)	0 (0.0)	1 (6.2)

*IMIDs* Immune-mediated inflammatory diseases, *INH* inhibitor

### Validation of anti-SARS-CoV-2 IgG testing

Positive IgG responses against the SARS-CoV-2 S1 domain were validated by two independent tests, one chemo-luminescence assay for IgG against the spike and nucleocapsid protein and an enzyme-linked immunosorbent assay for IgG against the nucleocapsid protein only (Fig. 1b). Furthermore, the pattern of immune responses against the spike protein S1 domain, the receptor binding domain of the S1 domain, the extracellular domain of the S2 domain and the nucleocapsid of SARS-CoV-2 were identical in the positively tested samples and patients with RNA proven COVID-19 but different from patients with endemic HCoV infection (Fig. 1b). These data indicate that anti-SARS-CoV-2 IgG responses are derived from COVID-19 but not endemic HCoV infections.

### Relation of anti-SARS-CoV-2 IgG to COVID-19 diagnosis

Notably, only 6 (13%) of the total 46 SARS-CoV-2 IgG positive participants received a diagnosis of COVID-19 during the observation period. This observation is in accordance with recently published data^9^ and also reflects the about tenfold difference between confirmed clinical COVID-19 cases in Bavaria (0.35%)^10^ and the seroprevalence of SARS-CoV-2 in this population study (2.2%). The difference in prevalence of confirmed clinical COVID-19 cases and seroprevalence of SARS-CoV-2 is based on several factors, which include (i) the availability of RNA testing, (ii) the sensitivity of RNA testing and (iii) the bias toward more symptomatic individuals being hospitalized and tested. The higher prevalence and broader range of symptoms in the anti-SARS-CoV-2 IgG positive participants with diagnosed COVID-19 than in those without diagnosed COVID-19 supports that notion (Supplementary Fig. S1).

### Exposure risk variables in IMID patients

To test whether differences in social exposure between the groups account for the low prevalence of SARS-CoV-2 IgG responses in IMID patients treated with cytokine inhibitors, we assessed exposure risk variables (contact with persons with a respiratory infection, presence at workplace outside home, travel to risk areas) of IMID patient groups and control groups.

**Fig. 2:**
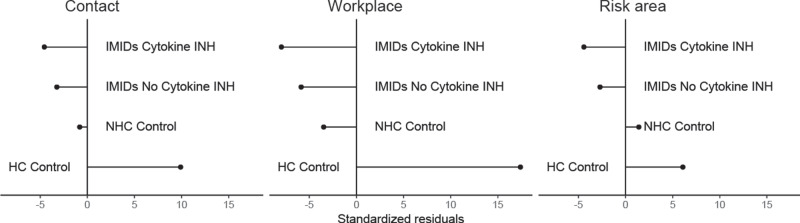
Exposure risk across study groups. Standardized residuals showing deviation from the expected frequencies for exposure risk variables (contact with persons with a respiratory infection, presence at workplace outside home, travel to risk areas) of IMID patient groups and control groups. A Pearson residual quantifies the individual contribution of each cell in a contingency table to the chi-squared statistic of the table and is calculated by subtracting the expected count in a cell from the observed count and dividing the result by the standard error. A Pearson residual is 0 when the observed cell frequency is equal to the expected and deviates from 0 accordingly as the observed cell frequency is greater or less than the expected count.

The deviation from expected frequencies of social contacts and behavior of IMID patients with and without cytokine inhibitors were very similar (Fig. 2), while, not unexpectedly, participants in the HC control cohort showed a pattern of higher exposure risk and higher frequency of symptoms (Table 3).

**Table 3:** Infectious symptoms.

	Non-healthcare control	Healthcare control	IMIDs cytokine INH	IMIDs non-cytokine INH
Symptom, *N* (%)	971	285	534	259
New musculoskeletal pain	68 (7.0)	19 (6.7)	57 (10.7)	31 (12.0)
Night sweats	59 (6.1)	31 (10.9)	46 (8.6)	37 (14.3)
Fever	58 (6.0)	15 (5.3)	26 (4.9)	15 (5.8)
Malaise/fatigue	94 (9.7)	68 (23.9)	87 (16.3)	36 (13.9)
Headache	216 (22.2)	97 (34.0)	119 (22.3)	44 (17.0)
Rhinitis	308 (31.7)	132 (46.3)	141 (26.4)	37 (14.3)
Shortness of breath	52 (5.4)	16 (5.6)	40 (7.5)	23 (8.9)
Cough	156 (16.1)	67 (23.5)	72 (13.5)	35 (13.5)
Throat pain	215 (22.1)	90 (31.6)	89 (16.7)	28 (10.8)
Anosmia	20 (2.1)	6 (2.1)	12 (2.2)	7 (2.7)
Diarrhea	77 (7.9)	29 (10.2)	85 (15.9)	25 (9.7)

*IMIDs* immune-mediated inflammatory disease, *INH* inhibitor

## Discussion

Our data are consistent with the idea that IMID patients treated with cytokine inhibitors show reduced susceptibility to SARS-CoV-2 infection and COVID-19. This finding is at first sight counterintuitive as IMIDs are associated with an inherently enhanced infection risk, also pertaining to viral infections^11^. Furthermore, inhibition of some of the pro-inflammatory cytokines has shown to increase the risk for infections, in particular bacterial and fungal infections^12^. Nonetheless, no increased risk for viral infections, i.e., respiratory infections such as influenza has been reported with the inhibition of TNF-α, IL-6, IL-17, or IL-23, which are the most frequent cytokine inhibitors currently used for the treatment of IMIDs^13^.

The low seroprevalence of SARS-CoV-2 in anti-cytokine treated IMIDs could have two principle explanations: While (i) the four groups were recruited in the same region, (ii) the HC control group having the highest prevalence for SARS-CoV-2 IgG was in direct contact with the IMID patients and (iii) all participants were exposed to similar detailed information regarding social behavior during the outbreak of the COVID-19 pandemic in Germany, IMID patients may have followed an even more stringent exposure prophylaxis than healthy individuals. We rigorously tested this hypothesis and showed that deviation from expected frequencies of social contacts and behavior of IMID patients with and without cytokine inhibitors was very similar, while, not unexpectedly, HC participants showed a pattern of higher exposure risk and higher frequency of symptoms. Since, seroprevalence of SARS-CoV-2 in IMID patients without cytokine inhibitors was similar to healthy participants, other factors need to be considered that account for this very low seroprevalence of SARS-CoV-2 in cytokine inhibitor-treated IMID patients.

Two recent studies reported that development of a more pronounced anti- SARS-CoV-2 IgG response is linked to more severe disease, suggesting inflammatory responses as important triggers for priming adaptive immunity against SARS-CoV-2^14,15^. Similar observations have been made for SARS-CoV^16^. Hence, tissue injury and the related release of proinflammatory mediators, which drive the symptoms of COVID-19, may also initiate robust antibody responses. Infection of epithelial cells by SARS viruses triggers the release of cytokines such as TNF-α and IL-6^17^, which are key cytokines in IMIDs, where they are known to aggravate inflammation and tissue damage. Therefore, treatment with cytokine blockers may dampen the inflammatory tissue damage in response to SARS-CoV-2, limit clinical onset of COVID-19 and also inhibit the formation of anti-SARS-CoV-2 antibodies. Not surprisingly, cytokine blocking strategies, such as IL-6 inhibition, are currently tested for the treatment of the hyper-inflammatory syndrome associated with COVID-19^18^.

This study also shows that overall seroprevalence of SARS-CoV-2 in the general population in Germany is low (around 2%), even in Bavaria that had the highest number of COVID-19 cases in Germany. However, this low prevalence may not be generalizable to other places. Data from Geneva in Switzerland, which have been hit more severely by the coronavirus pandemic, show a seroprevalence of around 10% in the general population^10^. Even more so, preliminary data from hotspots of coronavirus infection like London and New York City indicate an overall seroprevalence of SARS-CoV-2 around 20% for the general population and, consistent with our data, even higher rates in healthcare workers^19,20^.

Although we have extensively validated anti- SARS-CoV-2 antibody responses it cannot be fully excluded that some of the responses are due to cross-reacting antibodies raised by endemic coronavirus infections. If this was the case our data would indicate that IMID patients treated with cytokine inhibitors also have a reduced susceptibility to infections with endemic coronaviruses. Furthermore, virtual absence and/or mild clinical course of clinical COVID-19 infection in IMID patients^21–24^ argues for reduced susceptibility of these patients to SARS-CoV-2 and supports the concept of a protective or at least mitigating effect of treatment with cytokine-blocking drugs.

In summary, these data suggest that patients with IMIDs receiving cytokine inhibitors may not at enhanced but at lower risk for SARS-CoV-2 infection compared with the general community and IMID patients not receiving such drugs. These data support a central pathogenic role of cytokines in COVID-19 and may argue against stopping cytokine inhibitor treatment in patients with IMIDs during the current SARS-CoV-2 pandemic. However, only preventive clinical trials and/or larger prospective, observational studies will be the ultimate approach to answer the question of treatment discontinuation.

## Methods

### Patients

Patients with immune-mediated inflammatory diseases (IMID; *N* = 534) were recruited at the FAU Erlangen-Nuremberg, the Deutsches Zentrum fuer Immuntherapie (DZI), the Rheumatology Clinical Practice Erlangen, the Sozialstiftung Bamberg and at the Rheumatology Practice Bamberg. These centers are specialized in the treatment of IMID patients in the field of rheumatology, gastroenterology and dermatology. Patients were offered to participate if they were on stable (>3 months) treatment with cytokine inhibitors, comprising either therapeutic antibodies and receptors (such as those neutralizing tumor necrosis factor alpha or interleukins- 6, −17, or −23) or chemical agents (such as Janus Kinase inhibitors). In addition, a cohort of IMID patients (*N* = 259) receiving no cytokine inhibitors within the last three months was recruited in the same centers. Center distribution of IMID patients with and without cytokine inhibitors was identical.

### Healthy control cohorts

In addition, two control groups were analyzed. The first (*N* = 285) included health care (HC) professionals (doctors, nurses and technicians), who work in the aforementioned institutions and are involved in the treatment, diagnostics and research on IMID patients. Participants were contacted personally as well as by e-mail and invited to participate in the study. None of HC professionals denied participating in this study as their interest in receiving coronavirus diagnostics was very high. In addition and since the exposure of the HC control to infectious agents may be higher than in the general population, an additional control group with healthy participants not involved in health care was analyzed (“non-health care control”, NHC; *N* = 971). This group was composed of two populations: A cohort of healthy participants (*N* = 336) from the district of Erlangen-Höchstadt and the city of Erlangen, that has been established via field campaigns to assesses healthy ageing. These participants were not allowed to have a diagnosis of any IMID. Part of this cohort has been described previously^5^ and numbers have been expanded since then. The second part was a cohort of firefighters (*N* = 635) from the same region (district of Erlangen-Höchstadt and the city of Erlangen), which was recruited by an organized field campaign in March and April 2020. Recruitment was centrally organized by the volunteer fire brigade officers, who invited all firefighters of their sub-units to participate (via the corresponding fire brigade mailing lists). All firefighters agreed to participate in this study. As the prevalence of anti-SARS-CoV-2 antibodies was very similar in the non-firefighter cohort (2.12%) and the firefighter cohort (2.36%), the two NHC control cohorts were pooled.

### Ethical approval

Ethical approval (#157_20 B) to conduct this analysis was granted by the institutional review board of the University Clinic of Erlangen as the responsible ethics committee for all participating institutions. Written informed consent was obtained from the study participants.

### Demographic characteristics

Age, sex and body mass index were documented in all patients and healthy participants. In addition, history of smoking, arterial hypertension, diabetes mellitus, and chronic lung diseases, which put patients at risk for severe COVID-19 infection were documented (Table 1).

### Assessment of symptoms and social contacts

The study groups were exposed to questions on clinical symptoms (cough, rhinitis, throat pain, fever, headache, fatigue, musculoskeletal pain, anosmia, shortness of breath and diarrhea) and social contacts (contact with infected individuals, travel to at risk areas, working outside home) provided by the German federal authority for infectious diseases (Robert Koch Institute) and disseminated through routes of public communication. The following questions were asked to the participants (Fig. 2): (i) Contact with infected individuals: Have you had contact with people who had a feverish respiratory infection during the last 8 weeks? (ii) Travel to risk areas: Have you been in a risk area (e.g. Northern Italy, Tyrol) in the last 8 weeks or have you had direct contact with a confirmed or suspected case of COVID-19? (iii) Working outside home: Have you been working in the home office during the last 8 weeks? Data about symptoms and social contacts were self-documented (present/absent, complied/not-complied) using a standardized form during the study period (1st February to 15th April, 2020). These data were self-documented and retrieved when serum analysis was done after 8 weeks.

### Anti-SARS-CoV-2 antibody testing

The principle of testing IgG antibodies against the spike protein of SARS-CoV-2 has been described previously^25^. Serum samples were taken between March 18th and April 30th for anti-SARS-CoV-2 IgG tests. IgG antibodies against the S1 domain of the spike protein of SARS-CoV-2 where tested by the recent CE version (April 2020) of the commercial enzyme–linked immunosorbent assay from Euroimmun (Lübeck, Germany) using the EUROIMMUN Analyzer I platform and according to the manufacturers protocol. Optical density was determined at 450 nm with reference wavelength at 630 nm. A cutoff of ≥0.8 (OD450 nm) was considered as positive. This assay has very high (>99%) specificity (see also https://www.ukbonn.de/ C12582D3002FD21D/vwLookupDownloads/Streeck_et_al_Infection_fatality_rate_of_SARS_CoV_2_infection2.pdf/%24FILE/Streeck_et_al_Infection_fatality_rate_of_SARS_CoV_2_infection2.pdf) and has been approved for diagnostic use in the United States (see https://www.centerforhealthsecurity.org/resources/COVID-19/serology/Serology-based-tests-for-COVID-19.html#sec2). Assays were performed in line with the guidelines of the German Medical Association (RiliBAK) with stipulated internal and external quality controls.

### Tests used for validation of positive results

Two reference tests for IgG antibodies against the SARS-CoV-2 were used, one of them a chemo-luminescent assay for detecting antibodies against the spike and nucleocapsid proteins (Shenzhen Yhlo Biotech, iFlash-SARS-CoV-2, Cat #C86095G, Shenzhen, China). This assay is based on magnetic beads coated with SARS-CoV-2 spike and nucleocapsid antigen for the detection of specific IgG using a fully automated iFlash Immunoassay Analyzer (Shenzhen Yhlo Biotech). The assays were performed according to the manufacturer’s protocols. The IgG titer was automatically calculated as arbitrary units (AU/ml) and the cutoff value for a positive test was 10 AU/ml. In addition, an enzyme–linked immunosorbent assay detecting antibodies against the nucleocapsid protein (Immundiagnostik, Bensheim, Germany). Optical density was determined at 450 nm with reference wavelength at 630 nm. A cutoff of ≥0.5 (OD 450 nm) was considered as positive.

### In-house SARS-CoV-2 ELISA

An in-house ELISA was prepared according to published coating protocols^24^. In detail, 100 µl of the coating solution, consisting of 1.5 µg/ml of one of the above indicated SARS-CoV-2 antigens in 1xPBS, were applied to wells of a 96 well plates (Costar), incubated overnight at 4 °C. The following antigens were used: recombinant SARS-CoV-2 Spike Protein, S1 Subunit (1-Us-Tag); SARS-CoV-2 Spike S1 receptor binding domain (His-Tag) (both Sino Biological, Beijing, China); SARS-CoV-2 (2019-nCoV) Meridian Biosciences (Memphis, TX); Spike Protein (S2 ECD, His tag); SARS-CoV-2 (COVID-19) nucleocapsid protein(Sino Biological, Beijing, China). After removing the coating solution Liquid plate sealer animal free (#163 050, Candor, Germany) was added to the plates at room temperature for 1 h as blocking solution. Serum samples diluted 1:100 in 1% bovine serum albumin in 1xTBS/0.01% Tween were added to the plates for 30 min at room temperature. The following samples were used:^1^ Participants with anti-SARS-CoV-2 IgG negative IgG in the Euroimmun ELISA (*N* = 6)^2^, participants with anti-SARS-CoV-2 IgG positive IgG in the Euroimmun ELISA (*N* = 6)^3^, patients with COVID-19 infection and positive viral RNA test (*N* = 6), patients with endemic human coronavirus infection during the pre-SARS-CoV-2 era in spring 2019 (*N* = 5). Plates were washed three times with 200 µl per well of 1xTBS/0.01 % Tween. Next, a 1:15.000 dilution of goat anti-human IgG-horseradish peroxidase (HRP) conjugated secondary antibody (Dianova) was prepared in HRP-Protector solution (#222 050, Candor, Germany) and added to the wells for 15 min. Plates were again washed thrice with 1xTBS/0.01 % Tween. Once completely dry, 100 µl of 3, 3′, 5, 5′-Tetramethylbenzidine Liquid (TMB, Sigma Aldrich) Substrate solution was added to each well of the plates for 15 min and then the reaction was stopped by addition of 100 μL per well of 0.16 M sulfuric acid. Absorbance was measured at 450 nm. The positive cut off was equal to the mean of the OD values of the negative control wells on the respective plate plus three times the standard deviation of the OD value distribution from the negative control samples.

### Statistics

We summarized participant characteristics using means, standard deviations and percentages as appropriate. For anti-SARS-CoV2 IgG positivity (≥0.8 OD450 nm) exact 95% confidence intervals were constructed based on the Poisson approximation to the binomial distribution. We estimated relative risks of seropositivity in study groups using the NHC group as the reference and adjusting for age, sex, and sampling-date using a Poisson regression model with robust sandwich standard errors^25,26^. Age, sex, and sampling-date were included in the regression as they influence the risk for SARS-CoV-2 infection^9^. Adjustment for sampling date was achieved using the cumulative confirmed COVID-19 case-counts reported by the Robert Koch Institute for Erlangen and Erlangen-Höchstadt on the date of serum sampling (https://experience.arcgis.com/experience/478220a4c454480e823b17327b2bf1d4; accessed on 07.05.2020 at 16.24). We reasoned that these case counts would approximate the overall risk of exposure to SARS-CoV-2 from the onset of the pandemic to the date of sampling. We constructed 4 × 2 contingency tables for binary categories of (i) patient-reported history of contact with persons having a febrile respiratory tract infection, (ii) absence from workplace, such as working from home, being unemployed or retired, and (iii) travel to high-risk regions. Using these contingency tables we calculated and plotted standardized residuals showing the deviation of the observed frequencies in each study group from expected frequencies of the relevant category. Results for chemo-luminescent detection of anti-S1 spike protein antibodies (Yhlo Biotech) and anti-nucleocapsid antibodies (Immunodiagnostik) were compared between S1-positive and S1-negative participants using Mann Whitney *U* test. A *p* value of less than 0.05 was considered as significant. All analyses were done using R v.3.5.3 software (R Foundation for Statistical Computing, Vienna, Austria) and GraphPad Prism v8.1 (GraphPad Software, San Diego, USA).

### Reporting summary

Further information on research design is available in the Nature Research Reporting Summary linked to this article.

## Supplementary information

Supplementary information

Reporting summary

## Data Availability

Anonymized raw patient data have been deposited in GitHub under the 10.5281/zenodo.3929467 (https://github.com/ekoraytascilar/naturecommunicationscovid. All other data are present in the article and supplementary information files or available from the corresponding author upon reasonable request. Source data are provided with this paper.

## Source data

Source data

## Code availability

R/Sweawe code that reproduces the analyzes and tables reported in this manuscript are desposited in GitHub under the 10.5281/zenodo.3929467 (https://github.com/ekoraytascilar/naturecommunicationscovid). Source data are provided with this paper.
