# Supplementary material for: Patients with immune-mediated inflammatory diseases receiving cytokine inhibitors have low prevalence of SARS-CoV-2 seroconversion
Source: Nat Commun. 2020 Jul 24;11:3774. doi: 10.1038/s41467-020-17703-6 (PMC7382482; doi:10.1038/s41467-020-17703-6)
Supplement: Supplementary file 1 — Supplementary information [file 41467_2020_17703_MOESM1_ESM.pdf]

Supplementary Material

Simon D et al. Patients with immune-mediated inflammatory diseases receiving cytokine inhibitors have low prevalence of SARS-CoV-2 seroconversion.

Supplementary Figure 1. Symptoms in anti- SARS-CoV-2 IgG positive subjects with and without diagnosis of COVID-19.

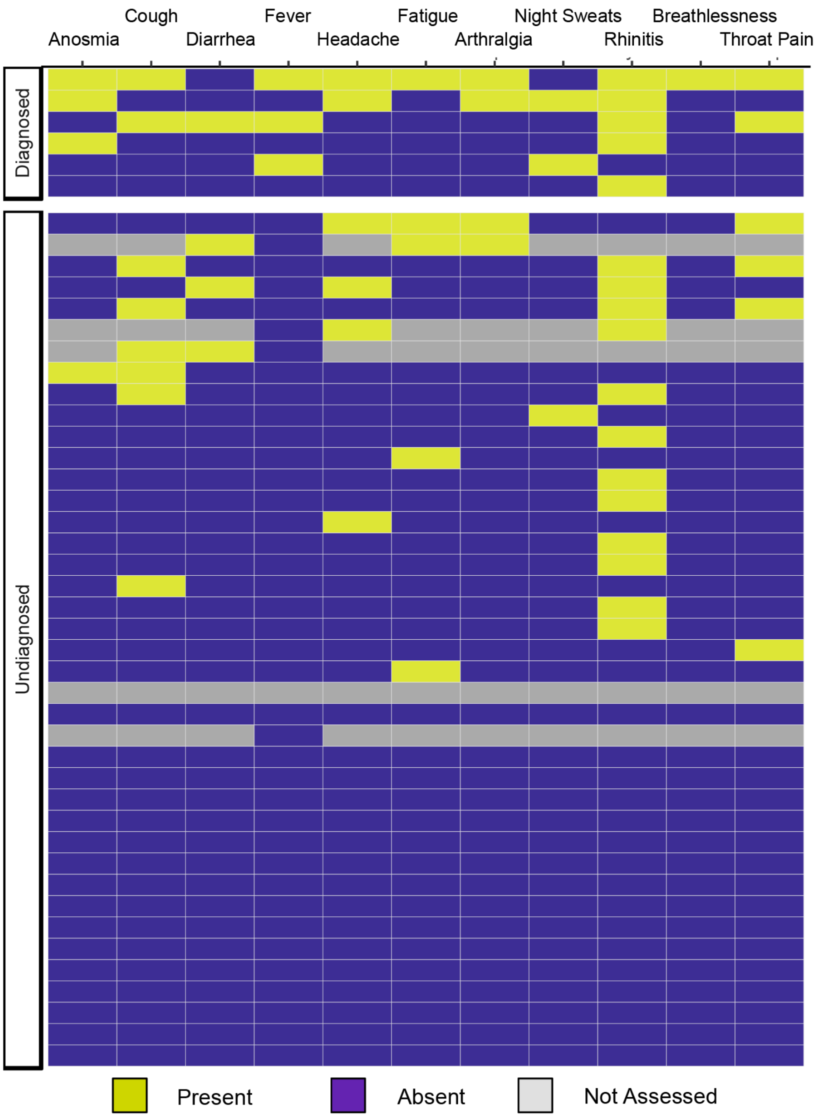

Legend. Chart showing infectious symptoms (columns) in the 45 subjects (rows) with a diagnosis of COVID-19 (upper part, "diagnosed") and without such diagnosis (lower part, "undiagnosed").
