# Supplementary material for: Patients with immune-mediated inflammatory diseases receiving cytokine inhibitors have low prevalence of SARS-CoV-2 seroconversion
Source: Nat Commun. 2020 Jul 24;11:3774. doi: 10.1038/s41467-020-17703-6 (PMC7382482; doi:10.1038/s41467-020-17703-6)
Supplement: Supplementary file 2 — Reporting summary [file 41467_2020_17703_MOESM2_ESM.pdf]

nature research

Corresponding author(s): Georg Schett

Last updated by author(s): Jul 2, 2020

Reporting Summary

Nature Research wishes to improve the reproducibility of the work that we publish. This form provides structure for consistency and transparency in reporting. For further information on Nature Research policies, see our Editorial Policies and the Editorial Policy Checklist.

Statistics

For all statistical analyses, confirm that the following items are present in the figure legend, table legend, main text, or Methods section.

n/a	Confirmed	
☐	☒	The exact sample size (*n*) for each experimental group/condition, given as a discrete number and unit of measurement
☐	☒	A statement on whether measurements were taken from distinct samples or whether the same sample was measured repeatedly
☐	☒	The statistical test(s) used AND whether they are one- or two-sided<br>*Only common tests should be described solely by name; describe more complex techniques in the Methods section.*
☐	☒	A description of all covariates tested
☐	☒	A description of any assumptions or corrections, such as tests of normality and adjustment for multiple comparisons
☐	☒	A full description of the statistical parameters including central tendency (e.g. means) or other basic estimates (e.g. regression coefficient) AND variation (e.g. standard deviation) or associated estimates of uncertainty (e.g. confidence intervals)
☐	☒	For null hypothesis testing, the test statistic (e.g. *F*, *t*, *r*) with confidence intervals, effect sizes, degrees of freedom and *P* value noted<br>*Give P values as exact values whenever suitable.*
☒	☐	For Bayesian analysis, information on the choice of priors and Markov chain Monte Carlo settings
☒	☐	For hierarchical and complex designs, identification of the appropriate level for tests and full reporting of outcomes
☒	☐	Estimates of effect sizes (e.g. Cohen's *d*, Pearson's *r*), indicating how they were calculated

Our web collection on statistics for biologists contains articles on many of the points above.

Software and code

Policy information about availability of computer code

Data collection	No software was used for data collection.
Data analysis	R 3.5.3, GraphPad Prism v. 8.1<br>Analysis code is publicly available through http://doi.org/10.5281/zenodo.3929467

For manuscripts utilizing custom algorithms or software that are central to the research but not yet described in published literature, software must be made available to editors and reviewers. We strongly encourage code deposition in a community repository (e.g. GitHub). See the Nature Research guidelines for submitting code & software for further information.

Data

Policy information about availability of data

All manuscripts must include a data availability statement. This statement should provide the following information, where applicable:

- Accession codes, unique identifiers, or web links for publicly available datasets
- A list of figures that have associated raw data
- A description of any restrictions on data availability

Anonymized raw data are publicly available at https://github.com/ekoraytascilar/naturecommunicationscovid under a Creative Commons BY 4.0 license (DOI: 10.5281/zenodo.3929467).

Field-specific reporting

Please select the one below that is the best fit for your research. If you are not sure, read the appropriate sections before making your selection.

- [x] Life sciences
- [ ] Behavioural & social sciences
- [ ] Ecological, evolutionary & environmental sciences

For a reference copy of the document with all sections, see nature.com/documents/nr-reporting-summary-flat.pdf

Life sciences study design

All studies must disclose on these points even when the disclosure is negative.

Sample size	In order to detect a 2% prevalence with 1% precision (half confidence interval length) 750 subjects would be required, our sample size of 971 healthy controls is more than sufficient for a precise seroprevalance estimate. In order to estimate half of this prevalence, 1%, with a 1% precision, 361 IMID patients under cytokine blockade would be needed, our sample size for the IMID group is 537. This could enable us to obtain even a more precise estimate and to demonstrate that a 2% prevalence in IMID patients under cytokine blockade is unlikely.
Data exclusions	No data were excluded.
Replication	Logistic requirements to obtain another 2000 samples to replicate our results would not be tractable.
Randomization	This is not an interventional study.
Blinding	Analyses of anti-SARS-CoV2 IgG antibody were performed by a technician, who was blinded to the identity and group allocation of the subjects. Other than the assays performed, data on respiratory infection symptoms and exposure risk behaviour were patient reported, for which blinding would not be possible.

Reporting for specific materials, systems and methods

We require information from authors about some types of materials, experimental systems and methods used in many studies. Here, indicate whether each material, system or method listed is relevant to your study. If you are not sure if a list item applies to your research, read the appropriate section before selecting a response.

Materials & experimental systems

n/a	Involved in the study	
[ ]	[x]	Antibodies
[x]	[ ]	Eukaryotic cell lines
[x]	[ ]	Palaeontology and archaeology
[x]	[ ]	Animals and other organisms
[ ]	[x]	Human research participants
[x]	[ ]	Clinical data
[x]	[ ]	Dual use research of concern

Methods

n/a	Involved in the study	
[x]	[ ]	ChIP-seq
[x]	[ ]	Flow cytometry
[x]	[ ]	MRI-based neuroimaging

Antibodies

Antibodies used	CE certified commercial test for anti-SARS-Cov-2 antibodies by EUROIMMUN
Validation	We used two reference assays to validate antibody positivity, a CLIA for detecting antibodies against the spike and nucleocapsid proteins (Shenzhen Yhlo Biotech, iFlash-SARS-CoV-2, Cat #C86095G, Shenzhen, China) and an enzyme–linked immunosorbent assay to detect antibodies against the nucleocapsid protein (Immundiagnostik, Bensheim, Germany). In addition we used in-house ELISA assays designed to detect a host of SARS-CoV-2 spike and nucleocapsid proteins in order to ensure that these components were also detectable in subjects positive with the primary assay and samples from individuals previously infected with garden variety Coronaviruses did not exhibit a similar antibody profile as those positive with the primary assay.

Human research participants

Policy information about studies involving human research participants

Population characteristics	Patients characteristics are described in Table-1 Data from overall 2049 subjects are presented: 534 with Immune-Mediated Inflammatory Diseases (IMID) receiving cytokine inhibitors, 259 with IMIDs receiving no cytokine inhibitors, 971 in the Non-Health Care Control and 285 in the Health Care Control group. The primary comparison adjusted for demographic differences between patient groups.
Recruitment	IMID patients: all IMID patients treated with cytokine inhibitors (group 1) or not being treated with cytokine inhibitors (group 2) attending the participating centers in Erlangen, Erlangen Höchstadt, Nuremberg and Bamberg between February 1st and March 15th. Health Care Controls: all physicians, nurses, technicians and laboratory personnel the participating centers, in which IMID patients were included Non-Health Care Control: (1) Consecutive cohort of healthy individuals with no link to the hospital and no IMID or other chronic disease; described in reference 5 in the manuscript; (2) all firefighters in the region Erlangen-Höchstadt. Subject recruitment did not involve a specific roster method or population sampling therefore individuals at higher risk of exposure to the virus that would be more willing to ascertain their immunity status against SARS-CoV-2 may have preferentially responded to the call. However we reason that although such preference could bias the seroprevalence figures upwards, it would effect all study groups in the same direction and be unlikely to introduce major bias into the relative effect estimates.
Ethics oversight	IRB of the University Clinic of Erlangen

Note that full information on the approval of the study protocol must also be provided in the manuscript.
